# Transcriptome sequencing and development of an expression microarray platform for the domestic ferret

**DOI:** 10.1186/1471-2164-11-251

**Published:** 2010-04-19

**Authors:** Carl E Bruder, Suxia Yao, Francis Larson, Jeremy V Camp, Ronald Tapp, Alexis McBrayer, Nicholas Powers, Willy Valdivia Granda, Colleen B Jonsson

**Affiliations:** 1 Southern Research Institute, 2000 Ninth Ave South, Birmingham, AL 35205, USA; 2 Orion Integrated Biosciences Inc, 265 Centre Ave, Suite 1R, New Rochelle, NY 10805, USA; 3 Center for Predictive Medicine For Biodefense and Emerging Infectious Disease, University of Louisville, KY 40292, USA

## Abstract

**Background:**

The ferret (*Mustela putorius furo*) represents an attractive animal model for the study of respiratory diseases, including influenza. Despite its importance for biomedical research, the number of reagents for molecular and immunological analysis is restricted. We present here a parallel sequencing effort to produce an extensive EST (expressed sequence tags) dataset derived from a normalized ferret cDNA library made from mRNA from ferret blood, liver, lung, spleen and brain.

**Results:**

We produced more than 500000 sequence reads that were assembled into 16000 partial ferret genes. These genes were combined with the available ferret sequences in the GenBank to develop a ferret specific microarray platform. Using this array, we detected tissue specific expression patterns which were confirmed by quantitative real time PCR assays. We also present a set of 41 ferret genes with even transcription profiles across the tested tissues, indicating their usefulness as housekeeping genes.

**Conclusion:**

The tools developed in this study allow for functional genomic analysis and make further development of reagents for the ferret model possible.

## Background

The ferret is an important model for pulmonary research studies because of the long trachea, large lung capacity and bronchiolar branching. It is commonly used for studies of infectious diseases and is susceptible to infection with a large number of pathogens, such as influenza virus, severe acute respiratory syndrome (SARS) corona virus, and canine distemper virus [1-8]. The ferret is an essential model for influenza research as it develops a number of the clinical symptoms of influenza that are also seen in humans and, in contrast to mice, can be infected by human isolates of influenza virus [9]. With the recent two-animal ruling by the US Food and Drug Administration (FDA) for the licensing of drugs or vaccines directed against diseases of low or no incidence, the ferret represents an inexpensive small, non-rodent animal model. Despite the fact that ferrets have been used in biomedical research for decades, little is known about the genome of *M. p. furo*. Currently, there are only a limited number of partial or full length cDNAs present in the GenBank. This not only impacts the number of molecular genetic assays available for analysis, but also hinders the development of antibody-based assays, such as flow cytometry. The lack of reagents for molecular analysis of the mechanisms involved in infection and immunogenic protection is restricted, limiting the usefulness of this animal model.

Recent advances in DNA sequencing have drastically increased the sequencing throughput relative to traditional Sanger based approaches [10-12]. Second-generation DNA sequencing technology enables more cost-efficient genomic analysis and, due to their highly parallel nature, allows for applications in transcriptomics and epigenomics. Sequencing of normalized cDNA libraries from several tissues allows for full or partial determination of the transcriptome of a model organism. The transcript information can then be used to develop microarray platforms, TaqMan assays and antibodies, for example [13-16].

Analyses of gene expression patterns provide important information on the role of differential gene expression in normal biological processes and disease progression. Global transcriptional changes detected using microarrays has improved our knowledge about virus-host interactions [17-19]. Examination of the mRNA levels in the host upon infection can help determine infection-specific transcriptional signatures and allow for distinction between different pathogens [20]. These functional genomic analyses may provide valuable knowledge about which components of the innate immune response the host activates to clear the pathogens, as well as the strategies that viruses or bacteria use to defeat these [21]. Identification of immune signatures can be used to assess the strength of the adaptive immune response and to predict protective immunity after vaccination [22]. To date, this type of analysis in the ferret is only possible through heterologous species array hybridization, which may introduce false positives due to the evolutionary distance between the target species and the microarray platform chosen (reviewed in [23]).

Here we report the use of massive parallel sequencing using the Roche GS-FLX platform to produce an extensive EST dataset for the ferret. To ensure sequence diversity, we pooled total RNA extracted from blood, liver, lung, spleen and brain. This RNA pool was reverse-transcribed to cDNA that was normalized prior to sequencing to avoid sequencing of abundantly expressed genes. The sequence reads were then assembled and annotated using a newly-developed annotation pipeline. The annotated reads were combined with ferret sequences already available in the GenBank, and used to create an oligonucleotide-based microarray. Our results contribute sequence information necessary for the development of molecular and genomic assays and allow for a better understanding of the genome of the domestic ferret.

## Results

### Transcriptome sequencing, annotation and sequence verification

To obtain a diverse representation of expressed sequences we pooled total RNA from blood, spleen, liver, lung and brain from one male and one female ferret. The RNA was then reverse-transcribed into cDNA. Prior to sequencing the cDNA was normalized to avoid the sequencing of only the most abundant transcripts.

We obtained approximately 104 million base pairs distributed in 518112 reads with an average read length of 201 base pairs. The reads were assembled into 54355 sequence contigs and annotated using nucleotide and/or protein sequence homologies. 33636 contigs were annotated. Initially, 32059 contigs were selected based on the assembled nucleotide length being at least 200 base pairs and the annotation e-value of 0.01 or lower (denoted as Ferret_seq1 here after). Based on the annotations, these contigs represented a total of 11707 ferret genes. All contigs in the Ferret_seq1 group were used for microarray probe design. The remaining contigs were megablasted against the available genomic ferret sequence as well as the HTGS and the NT databases available at the National Center for Biotechnology Information (NCBI). From these, 10710 contigs with sequence length larger than 100 bp and an e-value smaller than 10^-5 ^were selected to be included on the ferret-specific microarray (called Ferret_seq2 here after). The Ferret_seq2 cDNAs represent 4872 additional orthologous gene fragments not included in the Ferret_seq1 data set as well as 2374 hits to chromosomal regions.

**Figure 1:**
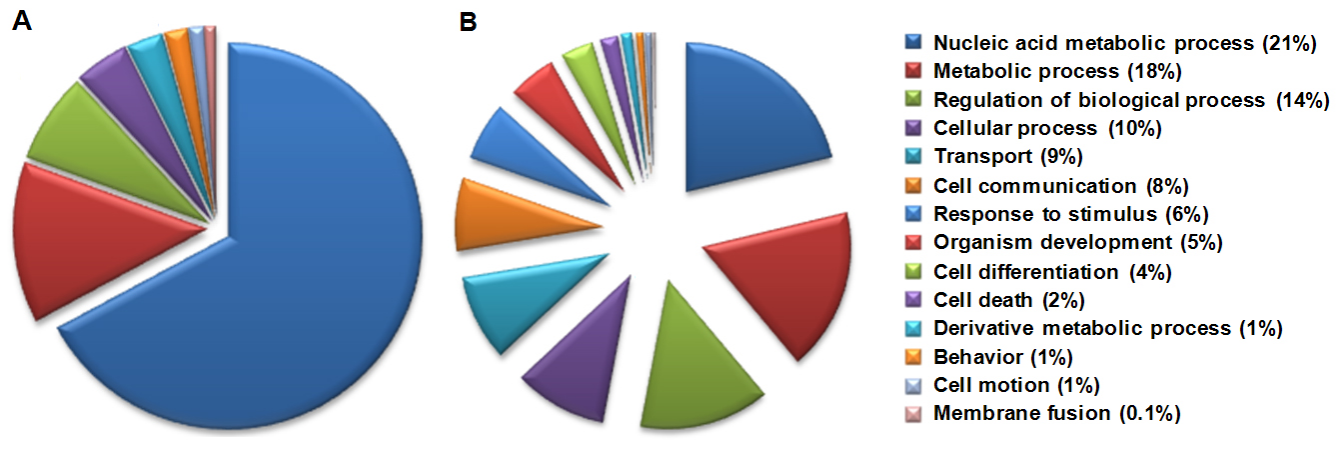
**Panel A shows a graphical representation of distribution of the contig-per-transcript coverage for the Ferret_seq1 and Ferret_seq2 sequence assemblies**. The majority of the transcripts were represented by a single contig (66%, in dark blue). 13% of the transcripts had two individual contigs covering parts of the gene (red). 7.5% of the genes were encompassed by three contigs (green), 4.5% of four contigs (purple), 3% by five contigs (turquoise), 2% by six contigs (orange), 1.3% by seven contigs(light blue) and 1% were covered by eight contigs. The genes covered by nine or more contigs represent approximately 2% when added together (not shown). Panel B shows the biological process distribution as per GO ontology analysis of the annotated contigs.

In an attempt to estimate the number of genes with multiple contigs covering distinctly different regions, we performed a reassembly of the Ferret_seq1 and Ferret_seq2 reads. Using less stringent CAP3 assembly settings, we identified 5951 contigs that were redundant and fully encompassed by other larger assemblies of sequences. These contigs were removed from further analysis. The remaining contigs were used to identify genes with multiple contig coverage. 3926 individual genes from the Ferret_seq1 and Ferret_seq2 are represented by contigs from two or more non-overlapping regions. The majority of those transcripts are covered by two individual contigs (15.9%). Approximately 6.9% of the transcripts from the ferret assemblies have three individual regions represented by sequence and approximately 3% are covered by four different regions (Figure 1A).

The annotated transcripts were also classified according to the three major functional standard Gene Ontology categories: biological process; molecular function; and cellular component. As expected, a wide range of functionality was covered. The most frequent biological processes are shown in figure 1b, and involve nucleic acid metabolic processes, metabolic processes and regulation of biological processes. The main molecular functions were binding, transferase activity, catalytic activity and transporter activity. Examining the cellular component annotations, more than 50% of the transcripts were classified as intracellular, approximately 30% were membrane proteins and only 6% were classified as extracellular transcripts (data not shown).

### Reduction of interfering alpha- and beta-globin mRNA in whole blood derived RNA

Phylogenetic analysis of the ferret globin genes indicated a closer relation to the human globins than to those of mouse. Using clustalw alignment of the sequences for the *HBA *gene for human, ferret and mouse resulted in higher alignment scores for *M. p. furo *versus Human (87 vs. 80 for ferret against mouse). We thus used the human GlobinClear ^® ^kit from Ambion to eliminate the globin interference and achieved an approximately 75% reduction of the globin mRNA (additional file 1). We further decreased the globin transcripts by complementing the human globin oligomix of the kit with ferret specific antisense RNA oligos directed towards the *HBA *and *HBB *genes, accomplishing an 85% reduction (additional file 1). We also hybridized blood-derived RNA with and without globin clear treatment on the ferret microarray. Hybridization of globin clear treated RNA increased the number of microarray probes with a normalized log_2_-transformed signal above 3 to 22112 probes versus 21438 for the non-treated RNA (data not shown).

### Design of the ferret microarray

We used the transcripts generated by the highly parallel sequencing described above, as well as the already publicly available sequences to create a microarray specific to *M. p. furo*. The array was designed utilizing the e-array portal hosted by Agilent for array design https://earray.chem.agilent.com/earray/. Using the quality scores assigned for each probe by the Agilent's probe design algorithm (denoted as BC score, with a range from one - high quality to four - low quality), we excluded all probes with BC scores of three and above. We further eliminated near-identical probes by blasting all probes against themselves. For probe pairs sharing 59 of 60 base pairs, only one probe was included on the array. We also included 4210 ferret entries in the EST database and 132 mRNAs were retrieved from the sequence repositories at NCBI. The ESTs were blasted against themselves to remove overlapping EST entries leaving 2270 entries. The mRNA and the ESTs were then uploaded to the e-array portal, and again probes with BC scores of three and higher were excluded from the array. Based on the annotation analysis, one or several different transcripts from 16644 ferret genes were represented on the array. In addition, the function of 2374 contigs remains to be elucidated as the blast hit used to annotate these points towards a genomic region rather than an expressed gene. The probe sequences, as well as the annotated source contigs or the gen bank accessions, are found in additional file 2.

### Microarray analysis and TaqMan development

**Figure 2:**
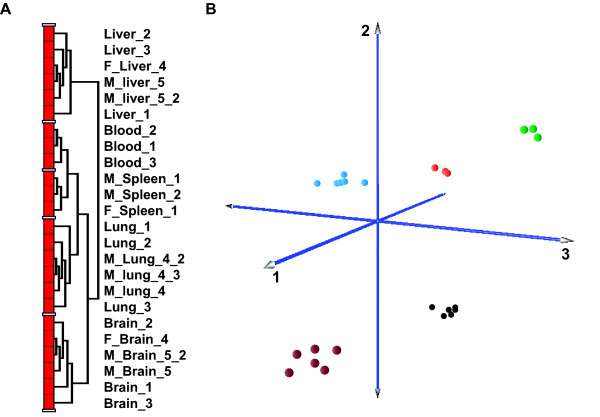
**Unsupervised hierarchical clustering and principal component analysis**. Panel A shows the hierarchical clustering of the different samples using all data points (Euclidian distance and average linkage clustering). Panel B shows the result from principal component analysis of the samples using all data points on the array. Maroon circles show the liver samples, black circles the brain samples, blue circles the lung samples, orange circles indicate the sample of spleen origin and green circles indicate the blood samples. Both of these methods separate the samples according to their biological origin.

RNA from brain, liver, lung, spleen and blood was analyzed on the array [GEO omnibus acc. GSE19398]. Biological replicates were analyzed as follows: five for liver and brain tissues, four for lung tissue, and three for blood and spleen tissues. In addition, technical replicates were analyzed for samples from the lung, the liver and the brain. 21350 probes of 43804 had a normalized, scaled and log_2 _transformed expression value above 2^3^, which was subjectively set as the low signal cutoff. The correlation coefficients between the biological replicates were 95% and higher. The technical replicates showed correlation coefficients of 98.5; 98.4 and 97.9% for lung, liver and brain respectively. Unsupervised hierarchical cluster analysis and principal component analysis using the entire data set was used to examine the power of the data to classify the samples according to their origin. Both methods showed a clear internal relationship between the biological and technical replicates from the individual samples (Figure 2). This is supported further by correct classification using K-nearest Neighbor classification analysis, where two samples in each sample group were selected to train the algorithm to classify the remaining samples (data not shown).

**Figure 3:**
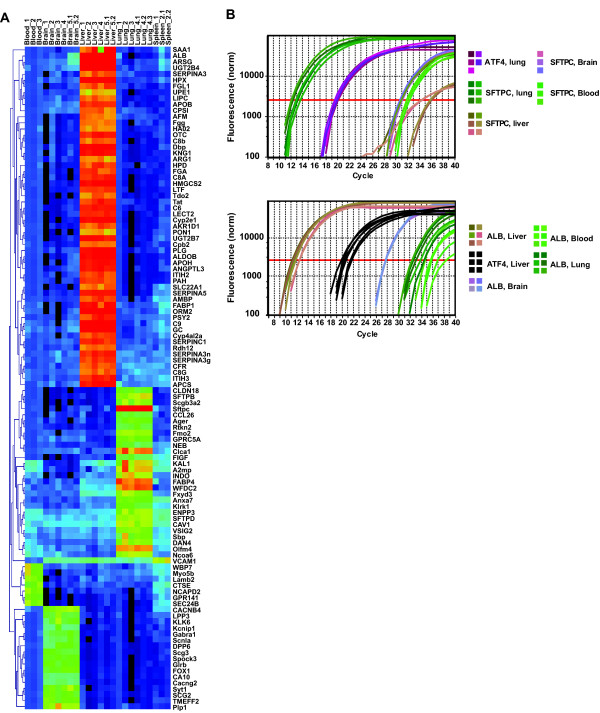
**Tissue-specific gene expression**. Panel A shows a selection of transcripts identified by ANOVA analysis followed by Pavlidis Template Matching analysis. The genes are clustered according to their expression profile for the analyzed tissues (Pearson correlation, average linkage clustering). The expression value is indicated by a sliding scale, going from black, indicating no expression values, through blue and green to red for the most highly expressed genes. Panel B show the tissue specific expression verification using qRT-PCR of two of the genes showed in panel A: the ferret albumin gene, *Alb*, and the ferret pulmonary surfactant-associated protein C, *Sftpc*. The graphs depict the log_2_-transformed curves of the relative fluorescence against cycle number during the qRT-PCR amplification. The analysis was done using RNA extracted from liver, lung, blood and brain, using the *Atf4 *housekeeping gene as reference. The C(t) threshold line (indicated by a red solid line) is set automatically using the noise band as cut off.

We then used parametric testing to isolate transcripts differentially expressed between the tissues. The one-way ANOVA test (p-value distribution based on the theoretical F-distribution, p-value cut-off of 0.001 and Bonferroni correction for multiple testing) isolated 8783 probes with significantly different expression profile between the five analyzed tissues. These probes were further analyzed using the Pavlidis Template Matching (PTM) pattern recognition algorithm to isolate transcripts only expressed by one of the five tissue groups (Figure 3). A number of these transcripts were compared to the gene atlas expression sets though the BioGPS portal [24]. The expression profile of a selection of these genes was further verified by TaqMan analysis.

**Figure 4:**
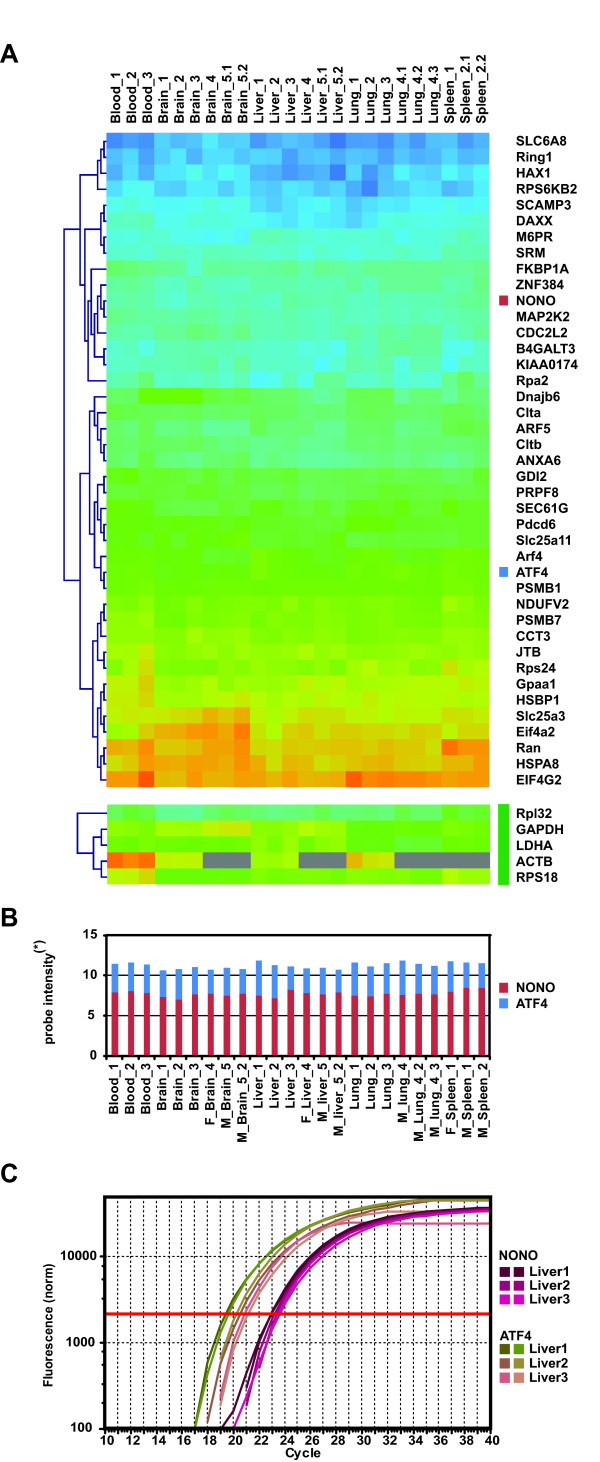
**Panel A: Hierarchical clustering analysis showing a set of possible housekeeping genes**. These genes were derived from the list of human housekeeping genes generated by Eisenberg and Levanon et al [24], and further selected using ANOVA analysis. The red and the blue bar indicate the *Nono *and the *Atf4*, shown in greater detail in Panel B. The green bar indicates the expression profile of some of the most commonly used housekeeping genes. Panel B: Normalized, scaled and log_2_-transformed probe intensities from *Nono *and *Atf4 *across the tissues. On average, there was a 3.57 fold difference between these two genes. Panel C shows the independent confirmation of the expression differences between the *Nono *and *Atf4 *in the liver. Quantitative RT-PCR determined *Atf4 *to have a 2.99 times higher expression than *Nono*, which resembled the array data well.

The array data were also used to identify housekeeping genes which had minimal variation across the tested tissues. We searched the array data set using the list of human housekeeping genes generated by Eisenberg and Levanon, 2003 [24]. The 609 probes on the ferret array corresponding to these genes were analyzed by ANOVA to filter out the genes that varied over the analyzed tissues despite being classified as housekeeping genes. More than 400 transcripts were significantly different (p-value distribution based on the 1000 permutations, p-value cut off of 0.001 and Bonferroni correction for multiple testing). The remaining transcripts were clustered using K-means clustering to distinguish between the genes that varied within each group from those that were truly non-variable between the groups. The latter indicate suitable housekeeping genes. We identified 41 genes from the Eisenberg and Levanon list with even expression across the tested tissues (Figure 4).

To verify a portion of the findings from the microarray analysis, we examined the gene expression differences between analyses by quantitative real time PCR. We identified five housekeeping genes with even expression levels across the samples. We also selected three genes per tissue, with the exception of the spleen, that were highly expressed only in the specific tissue. Primers and TaqMan probes were designed for each of the transcripts individually. We also designed primers and TaqMan probes enabling multiplex analysis, allowing for simultaneous analysis of tissue specific transcripts and housekeeping genes in a single reaction. Based on the theoretical optimal pairs combinations, the genes encoding apolipoprotein H (*Apo-H*, liver), albumin (*Alb*, liver), pulmonary surfactant-associated protein C (*Sftpc*, lung), Claudin-18 (*Cldn18*, lung), Glycine receptor subunit beta (*Glrb*, brain), Synaptotagmin-1 (*Syt1*, brain), ataxin 2-binding protein 1 (*Fox1*, brain), non-POU domain containing, octamer-binding protein (*Nono*, housekeeping gene) and lastly the activating transcription factor 4 gene (*Atf4*, housekeeping gene) were selected for analysis. We started the analysis by confirming the suitability of the two housekeeping genes. Both these genes showed even transcription profiles for the tested tissues. We then compared the relative expression differences between these genes. Based on the log_2_-transformed microarray data, the average difference of the fold-changes between the *Nono *and the *Atf4 *transcripts was approximately 3.5. Confirmatory analysis using qRT-PCR estimates this to be 3.0, which is in agreement with the microarray results (Figure 4). We also verified a number of the tissue-specific transcripts detected by the array analysis. The albumin and the pulmonary surfactant-associated protein C both showed low C(t) values, as expected. However, the fold-change ratio comparisons between the array data and qRT-PCR were higher when analyzed by qRT-PCR. A possible explanation is that the signal intensity of *Alb *and *Sftpc *genes is higher than the upper limit of the dynamic range of the microarray scanner. We then examined the *Fox1 *gene, exclusively expressed in the brain. This gene is expressed at lower levels than *Alb *and *Sftpc*, and the fold-change difference between *Fox1 *and *Atf4 *is approximately 1.5 when measured by the array and 0.5 when confirmed by qRT-PCR analysis (data not shown). Lastly, we tested several common housekeeping genes by real time PCR using SYBR green. These genes were the 60S ribosomal proteins L32 and L34 (*Rpl32 *and *Rpl34*) and the Sin3A-associated protein, 18 kDa (*Sap18*). As with the array analysis, the C(t) values across the tissues were more variable than the 41 selected by the ANOVA/K-means clustering analysis (data not shown).

## Discussion

### Lack of molecular and immunological tools

Domestic ferrets commonly serve as a model in critical research areas. The generation of reagents is critical to probing further into the mechanisms of disease and infection using this model. The ferret animal model is highly adapted for studies of influenza virus infection because the animals develop a number of the symptoms of influenza that are also seen in humans [4-8]. A severe course of disease is seen when infecting the ferrets with highly pathogenic strains such as the H5N1 avian influenza strains [3,25]. Ferrets can be infected by human isolates of influenza virus, while mice, for example, require adapted strains [9]. Humans and ferrets share the same molecular structure of sialic acid residues, which serve as the receptor for influenza attachment to the host cell in the airway epithelium. This enables the influenza virus to use the same host cell entry mechanism. In addition to influenza virus, the ferret model has been shown useful for studies of SARS CoV infection [1], Canine distemper virus [26], *Campylobacter sp*. infection [27] as well as in Alzheimer's disease research [28].

Despite the broad use of this animal model, ferret research has been hindered by a lack of available molecular genetic reagents and immunological tools. Current Entrez records show that 386 mRNAs, 4210 ESTs and 200 protein sequences are available. The ferret genome is not, as of yet, fully sequenced, however, the genome sequencing project is ongoing and has recently made several thousand partially sequenced clones available in the HTGS database. In addition, second generation sequencing technologies have increased the sequencing throughput significantly, and enable full or partial determination of the transcriptome of a model animal [13,16,29,30]. The goal for the present study was to utilize these advances to increase the available transcribed sequences for the ferret and to use this information to develop a ferret microarray platform as well as assays for quantitative real time PCR.

Analysis of global transcriptional changes using microarrays has improved our knowledge about virus-host interactions as well as mechanisms by which vaccines induce protection. Functional genomic analyses provide valuable knowledge about components of hosts' innate immune responses to pathogens, as well as the strategies the viruses or bacteria use to defeat these (our unpublished results and e.g. [17-19]). Systemic approaches like these can identify innate immune signatures; which may be used to assess the strength of the adaptive immune response and to predict protective immunity after vaccination [22].

Microarray analysis of RNA derived from ferret studies are commonly performed through heterologous array hybridization using an array developed for another species [19,31]. The sequence divergence between two different species may lead to influences on hybridization kinetics and the differentiation between actual differential gene expression and those that may be due to sequence mismatches become indistinguishable. In addition, isoforms and variants of ancestral genes may cross hybridize and give false positives (reviewed in [23]). The microarray presented in this work avoids these concerns.

### Transcriptome sequencing and ferret assay development

Sequencing of the normalized cDNA library resulted in more than five hundred thousand sequence reads. These reads were assembled, filtered and annotated using blast searches against the non-redundant protein and sequence databases. We identified more than 15000 individual transcripts, of which approximately 25% are covered by two or more contigs. The correctness of the annotation process was tested in several ways. We blasted contigs annotated based on protein sequences against the nucleotide databases. The annotations were also indirectly confirmed by the tissue-specific expression profiles detected on the microarray. The tissue-specific gene expression profiles were compared to the gene expression of the same genes from mouse and human using the BioGPS web portal http://biogps.gnf.org. The vast majority of the analyzed genes showed similar transcription profiles for the ferret genes and the human or murine transcripts. Common expression profiles suggest that the annotation of the ferret transcripts on the array is accurate. Although the possibility of altered expression of genes in the ferret, the human or the mouse exist, it is likely that the majority of the well-conserved expression patterns are similar between these species.

The microarray data were used to identify genes evenly expressed throughout the analyzed tissues. We based this analysis on a set of previously-identified housekeeping genes and identified 41 genes through ANOVA and K-means clustering analysis. The resulting genes had limited variation across the tissues. None of the most commonly used housekeeping genes, such as the Beta-Actin (*Actb*), Glyceraldehyde 3-phosphate dehydrogenase (*Gapdh*), or the 60S ribosomal protein L32 (*Rpl32*) were in this list. Analysis of the coefficient of variation (CV) among the data points across the tissue samples indicated several fold-higher CVs for these (*i.e*., the CV for *Actb *and *Gapdh *was 6.9 and 6.8% of the average log_2_-transformed expression signal, versus 2.7% for the *Atf4 *gene). Careful analysis of housekeeping genes is essential for both semi-quantitative and quantitative analysis of gene expression [32]. The 41 genes we present here provide a good starting point for further investigations into the ferret animal model.

## Conclusion

The ferret is an increasingly important animal model, especially since it is susceptible to infection of influenza strains that also infect humans. However, the number of reagents for molecular and immunological analyses is limited. In this study we have identified close to 16000 partial ferret genes, created a microarray platform for global transcription analysis, developed quantitative real-time PCR assays and hemoglobin mRNA reduction methods. The tools and sequences presented here can be used to aid in the development of ferret-specific antibodies, the discovery of molecular targets of pathogens in the ferret, infection-specific biomarker discovery and identification of correlates of protection for vaccine tests in this animal model.

## Methods

### RNA preparation, library preparation and sequencing

Four six-months-old healthy ferrets were anesthetized using a mix of ketamine, xylazine and atropine prior to the final blood draw and euthanasia. Ferret lung, liver, brain, and spleen tissue were taken and immediately snap-frozen in liquid nitrogen. Total RNA was isolated using TRIzol according to the protocol supplied by the manufacturer (invitrogen, Carlsbad, CA). Approximately 100 mg of frozen tissue was added to 1 ml of TRIzol and homogenized using an Omni TH homogenizer with disposable plastic generator probes. Blood samples were collected in PAXgene blood RNA tubes, and total RNA was extracted using the PAXgene Blood RNA extraction kit according to the supplier's protocol (PreAnalytiX GmbH, Switzerland). The quality of the total RNA was analyzed on the Experion automated gel electrophoresis system using the RNA StdSens Analysis Kit according to the protocol supplied by the manufacturer (BioRad, Hercules, CA).

The total RNA was then prepared for cDNA normalization and library construction by Eurofins MWG Operon (Huntsville, AL). In essence, cDNA was synthesized from a pool made from equal amounts of total RNA using a N6 random primer followed by ligation of the 454 adapters A and B to the 5' and 3' ends of the cDNA. The cDNA was then amplified by PCR using a proof-reading enzyme and assayed on a 1.5% agarose gel. The nucleotide fragments ranged from approximately 100 to 3000 base pairs in size. The cDNA was then normalized to reduce the likelihood of sequencing the most abundant transcripts only. Normalization was done by one cycle of denaturation and re-association of the cDNA. Re-associated double-stranded cDNA was separated from the remaining single stranded, normalized cDNA by passing the mixture over a hydroxylapatite column. The normalized cDNA was then amplified by PCR. After eight PCR cycles the normalized and amplified cDNA was then loaded onto a preparative agarose gel and cDNA ranging from 450 to 650 base pairs were excised from the gel. The cDNA was eluted from the gel using the NucleoSpin Extract II kit according to the manufacturer's protocol (Macherey-Nagel, Düren, Germany). The size-fractioned, normalized cDNA fragments were then subjected to 454 pyro-sequencing using the GS FLX Technology according to protocols supplied by the manufacturer (454 Life Sciences, Branford, CT). The sequences are available at the Transcriptome Shotgun Assembly (TSA) database with accession numbers EZ456440 - EZ516573.

### Sequence assembly and annotation

We implemented an annotation analysis system with five major components: (1) ESTs assembly system; (2) contig processing; (3) sequence homology analysis; (4) sequence annotation and (5) an object-relational storage and reporting tool. The sequence reads were assembled using the CAP3 algorithm[33]. The contig processing removed redundant sequences that were not included in the assembly process and had a length shorter than 150 bp. The resulting contigs were annotated by using a sequence homology analysis pipeline including blastx and blastn algorithms and local copies of protein and nucleic acid sequences available from the NCBI-NR, SWISS-PROT, the Kyoto Encyclopedia of Genes and Genomes (KEGG) and the Gene Ontology databases. The results from BLAST searches for each of these databases were parsed, stored and integrated in a relational database using different e-value cut-offs parameters. Contig hits with e-values larger than 0.01 from homology searches in different reference databases were discarded. The summary of the results was reported for each reference database using either validated genes or putative functions for predicted genes.

### Globin reduction

Blood contains a large proportion of globin mRNA transcripts, which may decrease the detection sensitivity of less abundantly transcribed genes on expression microarrays. Utilizing globin reduction methods can increase the sensitivity of whole blood-derived RNA. We used the GLOBINclear™ kit to decrease the effect of alpha and beta globin mRNA expression in total RNA preparations derived from whole blood (Ambion, Austin, TX). Initially, sequence comparisons of the globin A, B and E genes from ferret, human and mouse were done using clustalw at the EBI server http://www.ebi.ac.uk/Tools/clustalw2/index.html with default settings. These comparisons showed that the ferret globin transcripts were more similar to the human orthologs than to those of mouse. We thus used the human GLOBINclear ^® ^kit from Ambion to reduce the globin interference.

The partial ferret transcripts for alpha and beta globins were uploaded to SciTools antisense design tab found at the Integrated DNA technology DNA web portal http://www.idtdna.com. Using the default settings, including "all phosphorothioate backbone", we selected three antisense oligonucleotides with as similar Tm as possible. A biotin group was added at the five prime end of each oligonucleotide to enable the attachment of the streptavidin magnetic bead and subsequent globin removal. The sequences were as follows: HBA1: 5'Biotin-GCCCTACCCTTTCTTTCGG; HBA2: 5'Biotin-AGTAGGTCTTGGTGGTGGG; HBB: 5'Biotin-CAGCCTTCTCCTCACCAGT. These antisense oligos were diluted in nuclease-free water and pooled at a concentration of 400 ng/μl.

Five microgram total RNA was then hybridized with 1 μl of the human Capture Oligo mix or 1 μl of the human Capture Oligo mix and 400 ng (1 μl) of the ferret antisence oligo mix. The remaining procedure was carried out as specified by the manufacturer (Ambion, Austin, TX). Five hundred nanograms of the globin-reduced RNA were then used for cRNA synthesis using the MessageAmp kit according to the manual supplied by the manufacturer (Ambion, Austin, TX). The cRNA was then analyzed using the Experion RNA StdSens Analysis Kit according to the protocol supplied by the manufacturer (BioRad, Hercules, CA), and the raw data was exported into MS Excel for analysis and visualization (additional file 1).

### Microarray analysis

Five hundred nanograms of high-quality total RNA was labeled according to the one-color microarray-based gene expression analysis protocol supplied by the manufacturer (Agilent, Santa Clara, CA). Quantification and labeling efficiency assessment was done using the NanoDrop 2000 according to supplier's protocol (NanoDrop Products, Wilmington, DE). Samples with a cRNA synthesis yield below 1.65 μg and a specific activity less than 9.0 pmol Cy3 per μg cRNA were repeated. Labeled cRNA (1.65 μg) was then fragmented and mixed with the HI-RPM hybridization buffer and hybridized for 18-20 hours. The arrays were then washed according to the protocol supplied by the manufacturer (Agilent, Santa Clara, CA). The arrays were then scanned using a GenePix 4000B scanner (Molecular Devices, Sunnyvale, CA). Each slide was scanned at 5 μm resolution with two different PMT gain settings (450 and 480 V). The image files were then opened in Agilent's Feature Extraction software using the default Gene Expression protocol and the GE1_QCM_Feb07 quality control metric set. This software performed gridding, quality control and feature extraction. The raw data files were then opened in MS Excel ^® ^and the signal intensity (gProcessedSignal) was normalized by dividing all data points with the signal value of the 75th percentile of all of non-control probes (gPercentileIntensityProcessedSignal), as suggested by Agilent. The normalized expression data from each experiment was then copied into a multi-experiment spreadsheet and scaled using the average of the new spreadsheet containing the expression data from all signal values of the 75th percentile of the non-control probes, in accordance with the protocol from Agilent. Lastly, the normalized and scaled data was log_2 _transformed and imported in to the TIGR Multiexperiment Viewer (MeV) for further statistical analysis.

### Statistical analysis

MS Excel was used to calculate the correlation coefficients between the replicate samples based on the normalized, scaled and log2 transformed data. The statistical analysis of the microarray data was done in MeV. Initially, we performed unsupervised hierarchical clustering of the samples. The clustering analysis was done including all intensity values for all samples, using Euclidian distance and average linkage. We also used principal component analysis and K-nearest neighbor's classification to classify the samples, using the default setting in MeV. Selection of tissue specific gene expression was done using one-way ANOVA analysis using p-value distribution based on the theoretical F-distribution, p-value cut off of 0.001 and Bonferroni correction for multiple testing. This analysis was followed by Pavlidis Template Matching (PTM) pattern, manually setting the thresholds for the specific expression patterns. The housekeeping genes were selected using a gene list derived from Eisenberg and Levanon and identified through ANOVA analysis and K-means clustering. From the gene list we selected genes not significantly changed between the tissue groups. To sort out genes with large intra tissue variability we subjected the non-significant hits to K-means clustering and then manually selected the clusters with no or limited variability between the tissues. These genes were then hierarchically clustered using Pearson correlation (Figure 4).

### TaqMan analysis

For qRT-PCR analysis we started with 2 μg of the total RNA. The RNA was reverse transcribed into cDNA using SuperScript III, First-stand synthesis system for RT-PCR according to the supplier's protocol (invitrogen, Carlsbad, CA). The cDNA (1 μl) was then used in a 20 μl reaction, including 450 nM of each primer and 200 nM of the probe. We used TaqMan fast universal PCR Master Mix for our reactions. The qRT-PCR analysis was carried out in an Eppendorf RealPlex4S machine at 60°C annealing temperature. The probes were labeled on the 5' end with either the FAM or HEX fluorophores and with a black hole quencher 1 molecule on the 3' end. The analysis was made in the RealPlex software from Eppendorf, letting the software automatically set the threshold based on the noise band (Eppendorf North America, Westbury, NY). The primer and probe sequences were as follows: FOX1_F GGTTATGCCGCATACCGCTAC, FOX1_R GGAGCAAGTGTGTGGTGGTAG, FOX1_PR TGCCGCTGCCGCTGCCTA; ATF4_F TTTACCTTCCTGCAACCACTTC, ATF4_R TCATGGTAATGTAAGCAGTAGAGTC, ATF4_PR CTGTCCTCCACTCCAGATCATTCCT; ALB_F TGGTTTCATCTGCCAGAGAAAG, ALB_R CAAAGTCAGCTTTGGGGAATTTC, ALB_Pr TTCAAGTGTGCCAGCCTCCAGAA; SFTPC_F GCATCGCAGTGTATGACTATCAG, SFTPC_R AGAGCCTCAAGACTTGGGATG, SFTPC_Pr CTCCTGATTGCCTATAAGCCAGCCC. The PR suffix denotes the probe sequence.

## Abbreviations

EST: expressed sequence tag; NT: non-redundant NCBI nucleotide database; HTGS: High Throughput Genomic Sequences database; PMT: Photomultiplier tube.

## Authors' contributions

CEB and CBJ have designed the study, overseen the sequencing and written the manuscript. CEB designed and performed the microarray analysis. FL and WVG have developed the annotation pipeline, performed the bioinformatic analysis of the sequence data, and have written the manuscript. SY and NP have designed and carried out the real time PCR. JVC has been involved in the statistical analysis of the microarray results. RT and AB were involved in extraction and preparation of samples for sequencing and microarray analysis.

## Supplementary Material

Additional file 1 **Figure S1**. Reduction of interfering α and β globin in total RNA derived from whole blood. The graph shows the raw electropherogram as well as the absorbance values over time of cRNA. The distinct peaks at approximately 30 seconds show the globin mRNA band. The reduction of this band is most pronounced (85%) in the blood sample were a mixture of the commercial human globin reduction kit and two newly developed ferret antisense RNA oligos was used (green line in the line graph). Click here for file

Additional file 2 **Table S1**. Ferret transcriptome and microarray details. ID_REF - microarray probe identifier, GeneSymbol - gene name abbreviation, GeneName - full gene name, ProbeSeqeunce - the 60 base pair oligonucleotide constituting the individual microarray probe, ProbeType - sub-categorization of probes, depending on the source sequence. Ferret-Seq1 and Ferret_seq2 probes were generated from the parallel sequencing described here, Ferret_EST_GB and ferret_mRNA_GB sequences were obtained from NCBI. SourceSequence - the sequence used to design Ferret_seq1 and Ferret_seq2 category probes. SourceAccesion - accession number of the sequence used to design probes from the Ferret_EST_GB and Ferret_mRNA_GB Click here for file
