# Supplementary figures and images for: Transcriptome sequencing and development of an expression microarray platform for the domestic ferret
Source: BMC Genomics. 2010 Apr 19;11:251. doi: 10.1186/1471-2164-11-251 (PMC2873475; doi:10.1186/1471-2164-11-251)

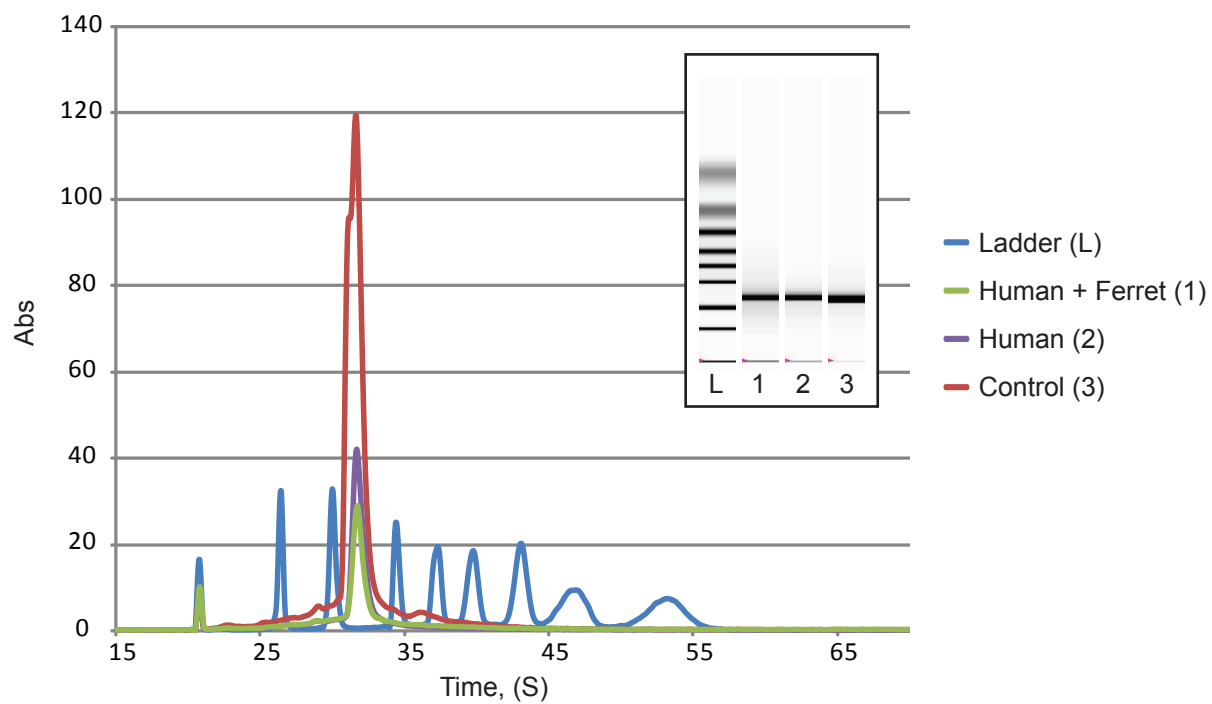

Supplement: Additional file 1 — Figure S1. Reduction of interfering α and β globin in total RNA derived from whole blood. The graph shows the raw electropherogram as well as the absorbance values over time of cRNA. The distinct peaks at approximately 30 seconds show the globin mRNA band. The reduction of this band is most pronounced (85%) in the blood sample were a mixture of the commercial human globin reduction kit and two newly developed ferret antisense RNA oligos was used (green line in the line graph). [file 1471-2164-11-251-S1.PDF]
